# Current status and challenges in timely detection of cervical cancer in Mexico: expert consensus

**DOI:** 10.3389/fonc.2024.1383105

**Published:** 2024-03-28

**Authors:** Salim Abraham Barquet-Muñoz, Cristina Arteaga-Gómez, Elsa Díaz-López, Amelia Rodríguez-Trejo, Janeth Marquez-Acosta, Carlos Aranda-Flores

**Affiliations:** ^1^ Department of Gynecological Oncology, National Cancer Institute, Mexico City, Mexico; ^2^ Department of Breast Tumors, National Institute of Perinatology, Mexico City, Mexico; ^3^ Colegio Mexicano de Especialistas en Ginecología y Obstetricia, Mexico City, Mexico; ^4^ Department of Gynecology, State Cancer Center, Nayarit, Mexico; ^5^ Colposcopy Department, Luis Catelazo Ayala Hospital, IMSS, Mexico City, Mexico; ^6^ Department of Oncology, General Hospital of Mexico, Mexico City, Mexico

**Keywords:** cervical cancer, HPV - human papillomavirus, screening, Mexico, prevention

## Abstract

Cervical cancer is a significant public health problem in low- and middle-income countries, accounting for 85% of new cases worldwide. Due to poorly organized screening programs, cervical cancer is more likely to develop in vulnerable groups who do not initiate or rarely undergo screening. Cervical cytology and detecting high-risk human papillomavirus types are the recommended screening tools. Further, these strategies allow for accurately identifying women at a higher risk of cervical cancer and establishing screening times. New detection tools, such as novel biomarkers or automatic HPV detection in the vagina or urine, can improve screening coverage. This review aims to identify the challenges faced by detection programs and screening tools in Mexico to provide evidence-based recommendations to improve early detection programs for cervical cancer.

## Highlights

- Cervical cancer remains a significant public health concern in low- and middle-income countries, accounting for a substantial proportion of new cancer cases and deaths worldwide. In poorly organized screening programs, vulnerable populations who do not participate or receive regular screening are at an increased risk of developing cervical cancer. The recommended screening tools for cervical cancer, supported by scientific evidence for routine use, include cervical cytology and testing for high-risk human papillomavirus (HPV). Additionally, various strategies have been explored to utilize these tools effectively to identify women at higher risk of cervical cancer and extend the intervals between negative screening tests with greater confidence. Ongoing research is evaluating innovative early detection methods, such as self-sampling for vaginal or urinary HPV testing, with the potential to improve screening coverage.
- Furthermore, studies investigate biomarkers that can enhance detection accuracy and provide more precise results. This consensus chapter aims to address the challenges associated with early detection programs and screening tools for cervical cancer in Mexico. It provides evidence-based recommendations to strengthen and improve early detection efforts, ultimately leading to better outcomes and a reduction in the burden of cervical cancer in the country.

## Introduction

Cervical cancer (CC) remains a significant public health concern, particularly in low- and middle-income countries, which account for 85% of its incidence (1). However, early detection programs can significantly reduce CC rates when national coverage exceeds 70% (2). In areas lacking organized screening programs, CC disproportionately affects vulnerable groups who rarely undergo or don’t start screening tests (3).

Early detection programs aim to identify asymptomatic women with precancerous lesions for treatment. The disparities observed in incidence and mortality among low-income countries can be attributed to unequal access and poor quality of screening tests. Therefore, enhancing screening programs requires the development of more sensitive, reproducible, and easy-to-perform detection tests (4). Cervical cytology has long been the primary screening method, but oncological high-risk HPV (HR-HPV) screening has gained increasing prominence (2).

This consensus chapter aims to identify opportunities for improving screening programs and tools and provide evidence-based strategies to enhance early detection programs for CC in Mexico.

## Limitations of screening programs in Mexico

### A. What challenges must be overcome by the timely detection programs for CC in Mexico?

Suba et al. establish three requirements for an adequate screening system for CC in low- or middle-income countries (5)

1. All women at risk should be screened.
2. All screening results must be accurate.
3. All women with abnormal results should receive appropriate management.

From the information provided, we can identify the challenges faced by the Mexican health system regarding CC. Unfortunately, there is a lack of recent evidence indicating the current status of CC within the Mexican National Health System. However, available primary evidence offers a general overview of the situation in the country (6). The Evaluation Report of the Cervical Cancer Prevention and Control Program in Mexico (2008–2011), prepared by the National Center for Gender Equity and Reproductive Health, highlights issues related to the quality of the timely detection system based on a database comprising 10,371,729 screening or follow-up tests. The report identified several challenges:

1. Low prevalence of cervical intraepithelial neoplasia (CIN2+) with cytology (0.98%).
2. High referral to colposcopy in patients with cytology with low-grade intraepithelial lesions (LIL) (44%).
3. High proportion of false negatives for high-grade intraepithelial lesion (HIL) in cytological diagnoses of LIL (6351 were CIN2+ and 197 were invasive).
4. High proportion of unnecessary biopsies in colposcopy (43/100 LIL by cytology).
5. High proportion of patients lost to follow-up with LIEAG, the most critical problem, since a complementary review was not performed in 49.9% of CIN2, 51% of CIN3, and 45.5% with a cytological diagnosis of carcinoma.
6. The country’s current conditions perpetuate logistical and operational problems (7).

In addition to medical factors, sociocultural and economic aspects influence non-attendance for cytology or follow-up. These factors have been studied in various settings, including Mexico. A study involving Mexican women aged 14 to 47 found that 43.8% had never participated in screening. Reasons for non-attendance included a lack of knowledge about cervical cancer, lack of interest, recent sexual debut, shame, or fear. Knowledge of HPV, cytology, and cervical cancer screening was low, with 50% having heard about HPV, 38.9% about cytology, and 25% about CC (8). In the northern region of Mexico, the situation is similar. An estimated 69,139 women should have been screened, but only 8,941 (12.9%) participated. Barriers identified by patients include lack of knowledge about navigating the local health system, low literacy, limited economic resources, inability to miss work, limited transportation, and fear of deportation (9). The COVID-19 pandemic has further complicated cervical cancer screening. During the pandemic, the number of cytology tests in Mexico decreased by 38%, although there has been a gradual increase since then (10). Follow-up appointments, including first-time and follow-up colposcopies, decreased by 9.1% and 10.6% annually, respectively (10, 11).

These findings highlight the need for targeted interventions to address sociocultural and economic barriers to cervical cancer screening and follow-up. Efforts should focus on increasing awareness about cervical cancer, improving access to screening, and providing support to women who may face challenges in navigating the healthcare system. Additionally, strategies to address the impact of the COVID-19 pandemic on cervical cancer screening and follow-up are needed.

#### Recommendation

A systematic and homogeneous registry is required for all health services to establish the current screening status in Mexico. The main challenges to be overcome by screening programs are socioeconomic difficulties, educational level, taboos, ignorance, and fear of the results. This group of women must be identified and apply for structured programs in their environment because they are vulnerable to not having follow-up. Quality of evidence: Moderate

### B. How has mortality from CC been modified in Mexico with early detection programs?

Despite the proven success of detection programs, there is a continued need to reduce both the incidence and mortality from cervical cancer (12). In May 2018, the Director General of the WHO proposed a strategy to eliminate cervical cancer. The goal of this strategy is for all countries to achieve and maintain a cervical cancer incidence rate of less than 4/100,000 women, with three key objectives:

1. Vaccinate 90% of girls up to 15 years old.
2. Screen 70% of women before age 35 and again after age 45.
3. Treat 90% of women with precancerous lesions and 90% of women with CC.

Each country must meet the 90–70–90 targets by 2030 to get on the path to eliminating CC (13).

In Mexico, initiatives to screen for cervical cancer have been implemented, and their impact has been measured. A study examining the incidence and mortality rates of CC in Mexico from 2000 to 2010 revealed 82,090 new cases and 46,173 deaths during this period. The incidence of new cases remained stable until 2007, followed by an 8.15% decrease, while mortality declined by 4.93% (14). Additionally, an estimate of CC mortality among women in Latin America and the Caribbean was conducted, with a prediction for 2030. During the period from 2014 to 2017, Mexico experienced a decrease in the average annual percentage variation (AAPC) of -3.9%, comparable to other countries such as Chile (AAPC: −2.4%) and Colombia (AAPC: −2.0%), among others. However, projections for 2030 indicate a rise in CC mortality due to alterations in population structure and size. Specifically, in Mexico, the predicted increase in incidence from 2014 to 2030 is from 25.0% to 26.5% per million annually, while the projected increase in deaths is from 756 to 981, representing an increase of 16.4% independent of population growth (15, 16).

#### Recommendation

Due to the implementation of national screening campaigns and the variation of public health programs, screening programs in Mexico have been partially successful and at different times. Timely detection programs in Mexico must adapt to the 90-70-90 strategy proposed by the WHO. Quality of evidence: Moderate

### C. What strategies can help improve the CC screening system in Mexico?

In Mexico, the National Health System offers organized CC screening programs to reduce the risk of cancer and death. These programs utilize cervical cytology and high-risk human papillomavirus (HR-HPV) testing (17). Additionally, models for ideal screening programs exist within Mexico. A study conducted at the Mexican Institute of Social Security found that HRVPH detection testing or co-testing (cytology with HR-HPV) in women aged 30 to 80 was more cost-effective than starting at age 20. Considering costs alongside false negatives, HPV detection was less expensive at USD 52.46 per detected case, identifying 93% of all CC cases. Co-testing was the next best option, costing USD 54.92 per case detected and seeing 98% of cases (18).

A study involving 36,212 Mexican women examined multiple strategies for screening cervical cancer. A comparison of six screening methods, comprising cytology alone, HPV 16/18 genotyping alone, and combinations of both, was conducted to determine their efficiency in detecting CIN2+. The sensitivity and specificity were calculated for the primary screening methods cytology (42.9% sensitivity and 74.0% specificity), genotyping (58.3% sensitivity and 54.4% specificity), and genotyping with reflex cytology (86.6% sensitivity and 34.0% specificity). The referral rate for colposcopy was twice as high with genotyping and reflex cytology (29%) compared to cytology alone (19). Additionally, a correlation was found between HPV 16/18 genotyping followed by reflex cytology and enhanced detection of CIN2+ compared to cytology alone (19).

Furthermore, a mathematical model study in the United States compared primary HPV detection starting at age 25 with cytology alone beginning at age 21 (20). Results indicated that primary HPV detection increased the detection of preventable cervical cancer by 13% and preventable deaths by 7% while yielding comparable years of life gained and only a modest 9% increase in colposcopies performed (20). These findings suggest that primary HRV HPV detection may be a more effective screening strategy compared to cytology alone, leading to improved early detection and prevention of cervical cancer. This approach warrants further investigation and consideration for implementation.

#### Recommendation

Screening with the HRVPH test is accurate and effective. Although the cost of cytology is not necessarily high, the cost of false negatives is. HRVPH could be a more cost-effective and appropriate alternative in early detection programs for national health programs. It is recommended to start screening with HRVHP from age 25 with 5-year intervals, or HR-HPV with cytology recheck every 5 years beginning at age 30. If you do not have an HPV test, perform cytology every 3 years. Quality of evidence: High

## Utility of screening tests for CC

**Table 1** shows the characteristics of the screening tests studied for the timely detection of CC.

**Table 1:** Comparison of diagnostic accuracy, advantages and disadvantages of early detection tools for cervical cancer.

	Sensitivity	Specificity	Advantages	Disadvantages	Cost	Reference
Conventional Cytology	30-87%	61-94%	It is simple to take, fix and read. Ideal for low income countries. You have a lot of experience.	It may be poorly preserved. It may have a reading error. Greater risk of inappropriate feedings. High variability in reading.	*	(1)
Liquid Base Cytology	55-87.5%	90-98%	Other molecular studies can be done. It can reduce inappropriate samples. There is much evidence about its use in screening. It is the most used currently.	Its negative predictive value is short-term. It has high variability.	*	(1)
HPV (DNA) test	89.7-98.4%	53.5-76.1%	There is much evidence about its role in screening. Can identify specific HPVs. Self-takes can be made.	Infrastructure is required. Low specificity in women under 30 years of age.	**	(2)
HPV (RNA) test	100%	75-84%	Negative predictive value lasts in the long term. Predicts progression.	Specific infrastructure is required. Long-term follow-up with negative cytology is unknown.	**	(3)
Dual staining	74.9-90.9%	72.1-95.2%	Predicts progression. Helps in diagnoses for cervical glandular lesions. Reduces unnecessary colposcopies.	Very specific applications. Requires training for interpretation. Requires specific infrastructure.	***	(4)
Methylation	64.5-94.7%	41.8-96%	Any type of sample can be used. Predicts regression or progression. The result persists in the long term.	It is still in the validation phase. The ideal variants are not known. Technology does not exist in primary care.	***	(5)

*, low ; **, medium; ***, high.

### A. What is the diagnostic accuracy of conventional and liquid-based cervical cytology screening methods for CC?

Cytology has consistently shown high specificity (about 98%) but variable and low sensitivity (about 55 to 80%). A meta-analysis assessing cytology reported a sensitivity of 30-87% and a specificity of 86-100% (21). The accuracy of cytology heavily relies on the adequacy of the sample. Sensitivity can be improved through training and techniques like using a cytobrush. For instance, meta-analyses revealed that the Ayre spatula is the least effective endocervical cell collection device compared to others (OR 2.25; 95% CI 2.06-2.44), with lower performance for dyskaryosis (OR 1.21; 95% CI 2.06-2.44). % 1.20-1.33) (22). Liquid-based cytology offers advantages such as improved collection and preparation, blood and debris filtering, and fewer unsatisfactory results. However, studies have shown no significant difference in sensitivity or specificity compared to conventional cytology. A cross-sectional study involving 800 patients compared conventional cytology, liquid-based cytology, and the HRVHP test for CIN2+ detection. Conventional cervical smears performed similarly (91% vs. 87%) (23). Thus, there is no evidence that liquid-based cytology reduces CC mortality compared to conventional cytology (24). Nevertheless, liquid-based cytology enables molecular identification of HRHPV.

#### Recommendation

Timely detection for CC can be performed with conventional or liquid-based cytology since it has the same diagnostic accuracy. However, the liquid base has the advantage that it can perform additional tests such as HRVHP detection and genotyping. If you do not have an HPV test, perform cytology every 3 years. The decision will be based on availability and clinical context. Quality of evidence: High

### B. What is the effectiveness of the HRVHP test as a primary detection method and as a co-testing to cervical cytology in the screening of CC?

The ATHENA study explored HR-HPV testing as a distinct screening procedure for CC. It involved 42,209 women over 25 years old. Cytology and HPV tests were performed. Women with unusual cytology or positive HPV were sent for colposcopy. The sensitivity for CIN3+ was considerably superior for HPV testing (76.1%) compared to cytology (47.8%; 95% CI 41.6-54.1%) or co-testing (61.7%; 95% CI 56.0-67.5%). However, specificity was marginally lower for HPV testing (93.5%; 95% CI 93.3-93.8%) than cytology (97.1%; 95% CI 96.9-97.2%) or co-testing (94.6%; (95% CI 94.4-94.8%)) (25). Another study with 19,009 women showed a lower cumulative incidence of CIN3+ at 48 months with primary HRVHP screening (2.3/1000) compared to cytology (5.5/1000) (26). A Californian cohort study with 990,013 women demonstrated reduced CIN3+ risk after consecutive rounds of negative co-testing (27). A follow-up study comprising 176,464 women found that HPV-based screening provided greater protection against CC than cytology.

A subsequent study involving four trials with 176,464 female participants between the ages of 20 and 64 revealed that screening using the HPV test provided 60-70% greater protection against CC than cytology. Comprehensive randomized trials suggest commencing HPV-based screening at age 30 and extending screening intervals to a minimum of 5 years (28). In the United States and other countries, primary HPV testing every 5 years is being explored as an alternative screening approach for CC. A mathematical modeling study was conducted to contrast cervical intraepithelial neoplasia grade 3 or worse (CIN3+) risks for three screening methods in a population of 1,011,092 women aged 30-64 with negative HPV testing and cytology. The findings revealed that 5-year risks after a negative HPV result were significantly lower than the 3-year risks after negative cytology results and 5-year risks after negative co-testing. The risk of CIN3+ was 0.069% after negative HPV compared to 0.19% after negative cytology (p<0.0001) and 0.11% after negative co-testing (p<0.0001). Similarly, the risk of cancer was 0.011% after negative HRVHP compared to 0.020% after negative cytology (p<0.0001) and 0.014% after negative co-testing (p=0.21). These findings suggest that primary HPV screening warrants consideration as another alternative to CC screening (29).

#### Recommendation

Co-testing (HRVPH detection and cytology) increases the sensitivity to detect LIEAG. The recommendation is to start it from 30 every 5 years if both are negative. Detection of HRVHP alone is more sensitive than cytology alone. Major international societies recommend focusing exclusively on primary HRVHP testing. Primary HR HPV testing is recommended every 5 years starting at age 25. It is recommended to use validated HPVHR tests in screening programs. (**Table 2**). Quality of evidence: High

**Table 2:** Characteristics of tests with scientific validity for the detection of HPV (6–9).

Types of HPV screening tests				Comments
**Tests based on the detection of HPV DNA**		**Direct genome detection**		
		1. Hybrid Capture II (HC2) (Qiagen Gaithersberg Inc., MD, EE.UU.)		Approved by the FDA in 1997 as a reflex test in patients with ASCUS cytology. Approved in 2003, as a Co Test in people over 30 years’ old Detects 13 high-risk types of HPV and 5 low-risk types. It does not determine the specific type
		2. Hybrid Capture II (hc2) (Qiagen Gaithersberg Inc., MD, EE.UU.)		Rapid test approved by the FDA, detects 14 types of high-risk HPV.
		3. careHPV (Qiagen Gaithersberg Inc., MD, USA)		Processes 90 samples of 2.5 hours.
		**HPV L1 fragment amplification**		
		1. Cervista HPV-HR (Hologic, Inc., Bedford, MA, USA)		Approved by the FDA in 2003 as a joint test, it analyzes High-Risk HPV DNA from 14 types. It cannot determine specific subtype.
		2. BD Onclarity HPV (Becton Dickinson, Sparks, MD, EE.UU.)		Approved by the US FDA, it detects types 16/18/45 specifically, as well as types 31/33/35/39/51/52/56/59/66/68.
		**Amplification and genotyping of HPV 16 and 18**		
		1. Cervista HPV 16/18 (Hologic Inc., Bedford, MA, USA)		Identifies subtypes 16/18, FDA approval. for joint testing in 2009
		2. Cobas HPV test (Roche Molecular Systems Inc., Alameda, CA, USA)		The only test currently approved for primary screening in the US starting at age 25. Reports specific results for HPV 16 and 18 and grouped results for types 31/33/35/39/45/51/52/56/58/59/66/68 Rapid test approved by the FDA, can process 96 samples in 5 hours
		3. Xpert HPV Cepheid (Sunnyvale, CA, USA)		Detects DNA encoding E6/E7 from 14 high-risk types. Each kit can process 10 samples per hour. Not approved by FDA
		4. Real-time high-risk HPV assay (Abbott Molecular, DesPlaines, IL, USA)		Simultaneous genotyping for HPV 16/18 and joint detection of 12 other HPV types. The response time is 6-8 hours, and it can process 96 samples in one cycle.
		5. PapilloCheck (Greiner Bio-One, Frickenhausen)		For qualitative detection and genotyping of 24 HPVs (13 high risk, 5 intermediate risk and 6 low risk)
**RNA-based HPV assays**		1. Aptima HPV Assay (Gen-Probe, San Diego, CA, USA)		Allows the detection of E6/E7 mRNA transcripts of 14 types of HPV
		2. Assay PreTect HPV-Proofer (Proofer NorChip)		Detects the mRNA of the E6/E7 oncogene of hr-HPV 16/18/31/33/45; high specificity for ASCUS cytology triage.
**Monoclonal antibodies.**		1. Avantage Assay HPV E6 (Arbor Vita Corporation)		The point-of-care test detects the E6 onco protein of HPV16/18/45/ 31/33/52/58. Useful for low-resource settings. Can process 45 samples in 2 or 2.5 hr.

There are currently more than 250 HPV tests on the market, only some are clinically validated (1).

Currently, only HC2 (Digene), cobas 4800, Aptima and BD Onclarity are clinically validated as primary tests for cervical cancer screening (2).

## Future perspectives on CC screening

### A. What are the future perspectives with self-collection and urine tests for timely detection of CC?

Various alternatives to conventional CC screening methods are currently under consideration for validation. One such alternative is the detection of HR-HPV using self-sampling methods, such as vaginal self-swabbing or urine collection. This approach offers several advantages. Firstly, it reduces inequity by making screening more accessible to individuals who may face barriers to accessing healthcare services, such as those living in remote areas or those with limited mobility. Secondly, self-sampling can provide samples for genotyping and measurement of biomarkers, which can augment risk assessment and guide personalized management strategies. The World Health Organization recognizes the potential benefits of self-sampling for improving global coverage of CC screening. However, the implementation of self-sampling should be guided by careful consideration of various factors, including the sociodemographic characteristics of the target population, appropriate testing intervals, the molecular platform used for HPV detection, and the classification system for HPV (16).

Meta-analysis studies have shown that self-sampling methods, particularly those based on PCR for HPV detection, exhibit comparable sensitivity to clinical samples in detecting cervical intraepithelial neoplasia grade 2 or higher (CIN2+; OR 0.99, 95% CI 0.97 to 1.02) (30). This suggests that self-sampling can effectively identify individuals at risk of CC.

Another alternative to conventional screening methods is the detection of HPV in urine. Although this approach is still in its early validation stages, recent studies have demonstrated promising results. For instance, the APTIMA hrHPV E6/E7mRNA assay has shown favorable performance detecting HRVHP in urine samples. Further research is required to validate the accuracy and feasibility of urine-based HPV detection as a screening modality (31).

#### Recommendation

Vaginal or urine self-sampling could offer an opportunity to play an essential role in improving coverage for CC detection. Still, they need to be recommended practices for screening in CC. Quality of evidence: Moderate.

### B. What is the future of other biomarkers in CC screening?

The identification of biomarkers for CC has paved the way for the development of personalized medicine approaches. Integrative analysis at different levels of cellular function provides valuable insights into cancer biology and the intricate interactions of biomolecules. Among the known markers, p16 and Ki-67 have demonstrated promising results. A meta-analysis confirmed the high specificity of p16 and p16/Ki-67 immunocytochemistry for low-grade intraepithelial lesions (LIL) (32). However, limitations include excessive detection, high costs, and potential adverse events (32). Other potential biomarkers include the detection of E6/E7 proteins or methylation. The expression level of E6/E7 mRNA increases with the degree of malignancy of LIL (33). Moreover, studies have shown that increased viral methylation of the HPV genome is associated with premalignant and malignant lesions (34). These findings suggest the potential of these biomarkers in predicting premalignant lesions and guiding targeted therapeutic interventions.

#### Recommendation

Biomarkers such as dual staining (p16/Ki-67), E6/E7 proteins, and methylation are technologies that do not have sufficient evidence to support their incorporation into screening programs and should only be used in a research setting. Quality of evidence: moderate -low.

## Author contributions

SB-M: Writing – review & editing, Writing – original draft. CA-G: Writing – review & editing, Writing – original draft. ED-L: Writing – review & editing, Writing – original draft. AR-T: Writing – review & editing, Writing – original draft. JM-A: Writing – review & editing, Writing – original draft. CA-F: Writing – review & editing, Writing – original draft.
